# Caregiving-related strain among informal caregivers of older adults with dementia: findings from a nationally representative study

**DOI:** 10.3389/fpubh.2025.1618379

**Published:** 2025-10-31

**Authors:** Sultan A. Shubair

**Affiliations:** Social Studies Department, King Saud University, Riyadh, Saudi Arabia

**Keywords:** family caregivers, unpaid caregivers, strain, Alzheimer’s disease, related dementias

## Abstract

**Background and objectives:**

Informal caregivers (ICs) of older adults with dementia experience caregiving-related physical, emotional, and financial strain. Little is known about their characteristics and caregiving-related strain differences by dementia status.

**Research design and methods:**

A cross-sectional study was implemented among probable, possible, and non-dementia ICs of older adults from the 2017 National Health and Ageing Trend Study and linked to the National Study of Caregiving data for a nationally representative sample of 2,652. Analysis of variance was used to investigate differences in characteristics and caregiving-related strain by dementia status.

**Results:**

ICs of older adults with possible dementia were more likely to report an income ≤$99,999 (97.2%, *p* < 0.001) than ICs of older adults with probable dementia (94.8%) or non-dementia (86.9%), with no other group characteristic observed. Caregiving-related strain varied significantly by dementia status (*p* < 0.001), with ICs of older adults with probable dementia reporting the highest physical, emotional, and financial strain compared to ICs caring for possible or non-dementia older adults.

**Discussion and implications:**

Dementia ICs face disproportionately higher strain and greater financial vulnerability, underscoring the need for targeted interventions such as respite care, financial support, and caregiver training to sustain caregiver well-being as dementia prevalence rises.

## Introduction

The number of older adults aged 65 or older in the U. S. was 58 million in 2022. By 2050, this number is anticipated to increase to 82 million. As the number of older adults increases, the number of cases of dementia will increase, as age is one of the risk factors for developing dementia. Nearly 6.9 million Americans aged 65 and older were diagnosed with dementia in 2024, a neurodegenerative disease that leads to gradual declines in cognitive, emotional, and physical functional abilities (1). These declines lead older adults with dementia to rely on others for caregiving. Some rely on formal caregivers, care providers associated with formal social services, or the healthcare system, whether volunteer or paid employees (2). However, 83% of the assistance for older adults in the United States comes from ICs, who generally are family members, relatives, and friends providing unpaid care for ill family members with chronic diseases such as dementia (1, 2).

The choice to care for ICs with dementia is influenced by various reasons, such as the desire to keep them at home and the sense of obligation (3). While caregiving for older adults with dementia is slightly similar to caregiving for individuals with other chronic diseases, caregiving for older adults with dementia tends to be more extensive. For instance, ICs for older adults with dementia are more likely to provide their care recipients with medical care and personal assistance than caregivers of individuals without dementia. They are also more likely to support their care recipients in activities of daily living (ADLs), multiple instrumental activities of daily living (IADLs), mobility, financial management, and advocacy than caregivers of individuals without dementia (1).

However, caregiving can be rewarding for some ICs, which in turn assists them in remaining resilient to many negative psychological and physical consequences of caregiving (4). For instance, ICs may experience positive gains from caring for a relative with dementia, such as an increased sense of growth and personal achievement, increased meaning and purpose in life, increased feeling of gratification, strengthened relationships, and a level of mutuality between the caregivers and care recipients, increased the feeling of being supported by others and increased sense of satisfaction (5). These positive aspects of caregiving have been associated with the high level of self-efficacy of the caregiver after controlling for caregiver demographical characteristics, caregiver depression, and care recipient neuropsychiatric symptoms (6).

Although caregiving can be a rewarding experience for some ICs, it can be an extremely burdensome experience for others due to providing extensive care and support and dealing with cognitive status, functional status, and problematic behaviors of the care recipients for a long time with no formal support (7–9). Due to this extremely burdensome caregiving experience, ICs may experience caregiving-related physical, emotional, and financial strain. Experiencing caregiving-related strain is expected to threaten the health, the quality of life, and the ability of ICs to provide appropriate care and support to the care recipients significantly (10–13).

For instance, studies have found a significant association between caring for a relative with dementia and poor physical health, such as the high prevalence of cardiovascular issues, poor sleep patterns, low immunity, and obesity (14, 15). Indeed, according to a report from the Alzheimer’s Association, dementia ICs are more likely to report poor physical health than formal and other ICs of care recipients without dementia due to the extensive care assistance required to support care recipients with dementia (1).

Additionally, studies have associated caregiving for a relative with dementia with experiencing emotional and psychological issues, such as high levels of depression, anxiety, irascibility, stress, compassion fatigue, and social isolation, which are anticipated to increase the vulnerability to diseases and reduce the ability to provide high care quality to the patients (15–17). Additionally, the experience of emotional strain is more common among dementia ICs than the general population and other ICs for individuals with other diseases (18).

Moreover, dementia care costs almost $400.000 throughout a person’s lifetime in 2023. ICs bear 70% of the lifetime cost of care in the form of unpaid care and out-of-pocket expenses (1). Thus, it is not surprising that dementia ICs are more likely to experience financial consequences in the short and long term than the general population, which can threaten economic stability (19–24).

Moreover, previous studies have found a statistically significant relationship between experiencing caregiving strain and certain factors of the care recipient, such as old/young age, low level of education, high level of cognitive impairment, high level of physical function, low level of dependency, and poor ability to cope with daily living activities (25). Studies have also found a significant association between experiencing caregiving strain and the level of neuropsychiatric symptoms of the care recipient, such as depression, anxiety, aggression, and agitation (11, 32). In addition, studies have associated the type of dementia, the severity of dementia, and the duration of illness with experiencing caregiving strain (10). Studies have also found an association between experiencing caregiving strain and characteristics of ICs, including psychological factors (e.g., poor physical health, poor mental health, low level of quality of life, reduced ability to manage symptoms, and low religious coping skills), caregiving related factors (e.g., poor level of family functioning and high load of dementia care) and socio-demographical factors [e.g., being older, being female, being a member of minority groups, having a low level of monthly income and low level of education; (18, 32)].

While the previous studies have found a correlation between caregiving strain and socio-demographical factors of ICs, such as age, sex, race, ethnicity, income, and education (25), they have not identified the characteristic differences in caregiving-related physical, emotional, and financial strain among ICs for older adults with probable, possible, and no dementia. This review suggests that a variety of caregiving-related physical, emotional, and financial strains among ICs may likely account for differences in characteristics between ICs and the dementia status of the care recipient. However, the literature review did not yield any extensive studies investigating the differences in experiencing caregiving-related physical, emotional, and financial strain by dementia status and caregiver characteristics, and none of this research is based on nationally representative data of ICs for older adults.

Given the growing number of dementia cases associated with the estimated growth number of dementia ICs, caregiving-related strain, and challenges related to providing healthcare services and support for caregivers and care recipients, addressing the differences in experiencing caregiving-related strain among ICs is of particular importance (1, 26, 27). By using nationally representative data, the current study addressed the following questions: (1) Are there characteristic differences among ICs for older adults by dementia status? and (2) Are there differences in experiencing caregiving-related physical, emotional, and financial strain among ICs by dementia status?

## Methods

### Participants and procedures

This is a quantitative cross-sectional study that drew on data from a nationally representative survey and its linked caregiver survey, the National Health and Aging Trends Study (NHATS) and the National Study of Caregiving (NSOC), from Round 7. NHATS is an ongoing longitudinal study that started in 2011 and is sponsored by the National Institute on Aging (NIH) with cooperation from the Johns Hopkins University Bloomberg School of Public Health. NHATS includes a nationally representative cohort of adults aged 65 years and older who enrolled in Medicare. Two hours of in-person interviews are conducted annually with participants or proxy respondents to collect data on sociodemographic and health factors. The NSOC was conducted alongside NHATS and included a nationally representative cohort of informal caregivers of older adults who participated in NHATS. Thirty-minute telephone interviews were conducted to collect sociodemographic, health, and caregiving-related data from informal caregivers who help or have helped older family members, relatives, or friends with self-care, mobility, or daily activities related to health and function. The sample and dataset construction of NHATS and NHATS-linked NSOC surveys are explained in detail in prior work by Wolff et al. (28).

The current study sample included community-living Medicare beneficiaries, adults aged 65 years and older who receive help with daily self-care and mobility, and primary ICs, who provide the most hours of care for an older family member, relatives, or friends (28). Additionally, since the nature of care provided by informal caregivers is likely to differ from that provided by formal caregivers (29), older adults living in nursing homes or residential care facilities were excluded from this study. In total, the current study sample consisted of 2,652 older adults and their primary informal caregivers. The present study was reviewed by the University of Louisville Institutional Review Board (IRB# 19.1112) and approved via Expedited Review Procedure. Since the current research involves materials [data, documents, records, or specimens] collected by a prior study, it was classified under Category 5, which means that it has been granted a waiver of informed consent.

### Measures

#### Caregiving-related physical, emotional, and financial strain

Measures of caregiving-related physical, emotional, and financial strain were drawn from participants’ responses to the NSOC survey. The NSOC collects information on participants’ physical, emotional, and financial strains during telephone interviews by asking them to indicate whether they experience financial, emotional, or physical strain. Response options for this are dichotomized into “Yes” and “No.” The responses to this question were utilized to examine the differences in the caregiving-related physical, emotional, and financial strain.

#### Dementia status classification

Dementia status classification was drawn from the NHATS survey. NHATS used multiple methods to assess cognition, including self-report, proxy-reported assessment, and a cognitive test battery. The self-report includes asking the study sample to indicate whether a doctor has told them that they have dementia or Alzheimer’s disease. The validated proxy-reported assessment of dementia includes asking informal caregivers about the memory, temporal orientation, judgment, and function of their older family members if they are unable to respond. The cognitive test battery is designed to assess cognitive domains, including episodic memory, speed/attention, visuospatial function, linguistic abilities, and executive functions. NHATS used the previous methods to classify dementia status into probable dementia, possible dementia, and no dementia (30). The current study used this classification of dementia to determine the status of dementia.

#### Caregiver characteristics

The current study included several caregiver characteristics that have been identified in prior studies as significant factors contributing to caregiving-related physical, emotional, and financial strain (1, 11). These included age (0 = 20–59, 1 = 60+), sex (0 = male, 1 = female), education (0 = less than Bachelor’s, 1 = bachelor’s or more), and yearly household income (0 = $99,999 or less, 1 = $100,000 or more) race/ethnicity (0 = NH white, 1 = NH black, 2 = Hispanic, 3 = NH other). These demographic factors were drawn from the NSOC survey.

#### Analytic approach

To examine the characteristics differences among ICs for older adults across different dementia statuses (including probable dementia, possible dementia, and non-dementia), a series of statistical techniques was employed. Cross-tabulation was used to examine the distribution of demographic variables, including age, sex, and education, across the dementia status categories. To further analyze potential associations between caregiver strain and dementia status, an Analysis of Variance (r × k) was conducted to assess the relationship between dementia status and various strain indicators, highlighting whether caregiver strain varied significantly between the groups. All statistical analyses were performed using SPSS version 28.0.1.

## Results

### Characteristics of the sample, overall and by dementia status

Table 1 shows that the majority (54.7%) of the sample of 2,652 ICs were over 60 years old, including (68.2%) females, and (60.8%) had less than a bachelor’s degree. Additionally, (88.4%) reported a yearly household income of $99,999 or less, and (64.1%) were white Americans. Broken down by dementia status, 213 (8.0%) had provided care for individuals with probable dementia, 215 (8.1%) had cared for individuals with possible dementia, and 2,224 (83.9%) had cared for individuals with non-dementia. The ICs of individuals with possible dementia were more likely to report a yearly household income of $99,999 or less (97.2, *p* < 0.001), compared to the ICs of individuals with probable dementia (94.8, *p* < 0.001), and the ICs of individuals with non-dementia (86.9, *p* < 0.001). No differences were observed in other characteristics.

**Table 1:** Characteristics of the sample, overall and by dementia status.

**Variable**	**Overall** ***N* = 2,652**	**Probable dementia** ***N* = 213 (8.0%)**	**Possible dementia** ***N* = 215 (8.1%)**	**Non-dementia** ***N* = 2,224 (83.9%)**	***p*** ^**a**^
Age, *n* (%)					
20–59	1,202 (45.3)	95 (44.6)	99 (46.0)	1,008 (45.3)	0.956
60+	1,450 (54.7)	118 (55.4)	116 (54.0)	1,216 (54.7)	
Sex, *n* (%)					
Male	844 (31.8)	68 (31.9)	70 (32.6)	708 (31.8)	0.977
Female	1808 (68.2)	145 (68.1)	145 (67.4)	1,516 (68.2)	
Education, *n* (%)					
Less than Bachelor’s	1,613 (60.8)	129 (60.6)	131 (60.9)	1,355 (60.9)	0.995
Bachelor’s or more	1,039 (39.2)	84 (39.4)	84 (39.1)	869 (39.1)	
Yearly household income, *n* (%)					
$99,999 or less	2,345 (88.4)	202 (94.8)	209 (97.2)	1933 (86.9)	<0.001
$100,000 or more	307 (11.6)	11 (5.2)	6 (2.8)	291 (13.1)	
Race/ethnicity, *n* (%)					
NH white	1701 (64.1)	138 (64.8)	137 (63.7)	1,426 (64.1)	0.999
NH black	701 (26.4)	56 (26.3)	57 (26.5)	585 (26.3)	
Hispanic	159 (6.0)	13 (6.1)	13 (6.0)	133 (6.0)	
NH other	91 (3.5)	6 (2.8)	8 (3.8)	80 (3.6)	

NH: non-Hispanic. ^a^ Based on a chi-square test for categorical variables.

### Caregiving-related strain of the sample, overall and by dementia status

Table 2 shows that the vast majority of ICs (84.1%) had financial strain, while (78.7%) had physical strain, and (58.1%) had emotional strain. Broken by dementia status, the ICs for older adults with probable dementia were more likely to report caregiving-related physical strain (97.7, *p* < 0.001), compared to ICs for older adults with possible dementia (97.2, *p* < 0.001), and ICs for older adults with non-dementia (75.1, *p* < 0.001). Additionally, the ICs for older adults with probable dementia were more likely to report caregiving-related emotional strain (71.8%, *p* < 0.001) compared to ICs for older adults with possible dementia (71.6%, *p* < 0.001) and ICs for older adults with non-dementia (55.5%, *p* < 0.001). Additionally, the ICs for older adults with probable dementia were more likely to report caregiving-related financial strain (98.6%, *p* < 0.001) compared to ICs for older adults with possible dementia (98.1%, *p* < 0.001) and caregivers for older adults with non-dementia conditions (81.4%, *p* < 0.001).

**Table 2:** Caregiving-related strain of the sample, overall and by dementia status.

Type of caregiving-related strain	Overall *N* = 2,652	Probable dementia *N* = 213 (8.0%)	Possible dementia *N* = 215 (8.1%)	Non-dementia *N* = 2,224 (83.9%)	*p* ^a^
Physical strain, *n* (%)					
Yes	2087 (78.7)	208 (97.7)	209 (97.2)	1,670 (75.1)	< 0.001
No	565 (21.3)	5 (2.3)	6 (2.8)	554 (24.9)	
Emotional strain, *n* (%)					
Yes	1,541(58.1)	153 (71.8)	154 (71.6)	1,234 (55.5)	< 0.001
No	1,111(41.9)	60 (28.2)	61 (28.4)	990 (44.5)	
Financial strain, *n* (%)					
Yes	2,230 (84.1)	210 (98.6)	211 (98.1)	1810 (81.4)	< 0.001
No	422 (15.9)	3 (1.4)	4 (1.9)	414 (18.6)	

^a^ Based on a chi-square test for categorical variables.

## Discussion and implications

This study is the first to use nationally representative data to investigate how caregiver characteristics and caregiving-related strain vary by dementia status in a sample of ICs for community living Medicare beneficiaries’ older adults needing help with mobility and self-care. We examined whether there are characteristic differences among ICs for older adults with probable, possible, and non-dementia. Second, we examined whether caregiving-related physical, emotional, and financial strain differs among ICs for older adults with different dementia statuses.

No differences were found among the three groups of ICs in terms of age, sex, education, and ethnicity. Thus, the majority of probable, possible, and non-dementia cases were over 60 years old, female, had less than a bachelor’s degree, and were NH white. However, the yearly household income significantly differed among IC groups (*p* < 0.001). The vast majority of ICs for older adults with possible dementia (97.2%) and probable dementia (94.8%) reported incomes of $99,999 or less, compared to 86.9% of ICs for those without dementia. Additionally, caregiving-related strain varied significantly by dementia status (*p* < 0.001). ICs for older adults with probable dementia were more likely to report physical (97.7%), emotional (71.8%), and financial (98.6%) strain than those caring for individuals with possible dementia (97.2, 71.6, and 98.1%, respectively) or non-dementia (75.1, 55.5, and 81.4%, respectively).

Our finding that ICG of older adults with dementia, whether possible or probable, were more likely to report lower household incomes compared to ICG of individuals without dementia is consistent with prior research demonstrating the intersection between dementia caregiving and financial vulnerability. Both direct and indirect costs of care can undermine economic stability (19, 24). Limited financial resources may, in turn, reduce access to paid support services and increase reliance on uncompensated family care, thereby intensifying the strain on caregivers. Financial stress has also been associated with adverse psychological and physical health outcomes among caregivers, further compounding the already heightened demands of dementia care (31). These findings highlight the need for policy initiatives and support programs that address not only the clinical and emotional challenges of dementia caregiving but also the socioeconomic disparities that shape caregivers’ experience.

Our findings also revealed that caregiving-related strain varied significantly by dementia status (*p* < 0.001). ICs of older adults with probable dementia reported the highest prevalence of physical, emotional, and financial strain, followed closely by those caring for individuals with possible dementia. In contrast, ICG of older adults without dementia experienced notably lower levels of strain. These findings are consistent with prior research showing that ICs of individuals with dementia impose more extensive and complex responsibilities compared to ICs of individuals with other chronic illnesses (1, 3). Dementia caregiving often requires assistance with both activities of daily living (ADLs) and instrumental activities of daily living (IADLs), as well as medical care, mobility support, and financial management, which increases caregiver strain (1). As the U.S. population ages and dementia prevalence rises, with nearly 6.9 million Americans aged 65 and older diagnosed with dementia in 2024 (1), the proportion of ICs of individuals with dementia experiencing severe physical, emotional, and financial strain will likely continue to grow.

The elevated physical strain we observed aligns with evidence that dementia caregivers are more likely to suffer from poor health outcomes, including cardiovascular problems, sleep disturbances, and weakened immunity, compared to caregivers of older adults without dementia (14, 15). Emotional strain among dementia caregivers has also been widely documented, with studies linking caregiving to heightened risks of depression, anxiety, stress, and social isolation (16–18). Financial strain is another well-established challenge, with dementia caregiving estimated to cost nearly $400,000 over a lifetime, 70% of which is borne by families in the form of unpaid care and out-of-pocket expenses (1). Our findings reinforce these reports, as financial strain was especially prevalent among ICs of probable and possible dementia cases.

While some studies highlight that caregiving can yield positive outcomes, such as increased resilience, purpose, and strengthened relationships (4–6), the consistently higher strain levels we found among dementia caregivers suggest that the burdens often outweigh these benefits, particularly without adequate support systems. Taken together, our findings emphasize the urgent need for policies and interventions that deliver targeted support to dementia caregivers. As the number of dementia cases continues to rise alongside the aging U.S. population, expanding access to formal support services, respite care, financial assistance, and caregiver training will be essential to reduce the multidimensional strains faced by ICs and to sustain their ability to provide high-quality care.

Our analysis differs from earlier works in several key ways. First, it focuses specifically on the experiences of primary ICs, rather than all caregivers. This distinction is important because primary ICs are at greater risk of caregiving-related strain due to the substantial amount of time they devote to assisting older adults with mobility limitations and self-care disabilities. Second, unlike prior studies that often examined caregivers of older adults in nursing homes or residential care facilities, our study investigates ICs who support community-dwelling Medicare beneficiaries. The type of care provided in community settings differs substantially from that delivered by formal caregiving arrangements (28), making this a critical area of inquiry. Third, our study builds on previous research by examining variations in caregiving-related strain across different dementia statuses and caregiver demographic characteristics. These differences highlight important pathways for future research and underscore the need to design tailored interventions that address the heightened burden experienced by specific subgroups of caregivers.

## Limitations

Our study findings must be examined in the context of several methodological limitations. The study sample was drawn from the population of older adults aged 65 and older living in the community who received mobility and self-care disability assistance from primary caregivers. Thus, it is difficult to know to what extent our findings can be generalized to caregivers for older adults with dementia who are less than 65 years old, not living in the community, and without a disability. Also, our results cannot be generalized to formal and paid caregivers for older adults with dementia.

## Conclusion

This study is the first to utilize nationally representative data to investigate how caregiver characteristics and caregiving-related strain differ by dementia status among primary informal caregivers (ICs) of community-dwelling Medicare beneficiaries who require assistance with mobility and self-care. Our findings show that while demographic characteristics such as age, sex, education, and ethnicity did not differ significantly across caregiver groups, income disparities and experiences of caregiving-related strain were strongly associated with dementia status. ICs of older adults with probable and possible dementia were disproportionately likely to report lower household incomes and higher levels of physical, emotional, and financial strain compared to caregivers of non-dementia individuals. These results reinforce prior evidence that dementia caregiving entails more extensive and complex responsibilities than other forms of caregiving and highlight the compounding effect of financial vulnerability on caregiver well-being. With dementia prevalence projected to increase substantially as the U.S. population ages, the burdens experienced by ICs are expected to intensify, underscoring the urgent need for policies and interventions that target the multidimensional challenges of dementia caregiving. Expanding access to respite care, formal support services, financial assistance, and caregiver training will be essential to sustaining the capacity of ICs to provide high-quality care. Future research should broaden the scope to capture diverse caregiving contexts and explore strategies that address the socioeconomic inequalities shaping caregiver experiences.

## Data Availability

The original contributions presented in the study are included in the article/supplementary material, further inquiries can be directed to the corresponding author/s.
